# Effects of Magnitude of Leading Stimulus on Prepulse Inhibition of Auditory Evoked Cerebral Responses: An Exploratory Study

**DOI:** 10.3390/life11101024

**Published:** 2021-09-28

**Authors:** Yasuhiro Kawano, Eishi Motomura, Koji Inui, Motohiro Okada

**Affiliations:** 1 Department of Neuropsychiatry, Mie University Graduate School of Medicine, Tsu 514-8507, Japan; y-kawano@clin.medic.mie-u.ac.jp (Y.K.); okadamot@clin.medic.mie-u.ac.jp (M.O.); 2 Department of Functioning and Disability, Institute for Developmental Research, Aichi Developmental Disability Center, Kasugai 480-0392, Japan; inui@inst-hsc.jp

**Keywords:** change detection, MEG, N1m, prepulse inhibition

## Abstract

An abrupt change in a sound feature (test stimulus) elicits a specific cerebral response, which is attenuated by a weaker sound feature change (prepulse) preceding the test stimulus. As an exploratory study, we investigated whether and how the magnitude of the change of the prepulse affects the degree of prepulse inhibition (PPI). Sound stimuli were 650 ms trains of clicks at 100 Hz. The test stimulus was an abrupt sound pressure increase (by 10 dB) in the click train. Three consecutive clicks, weaker (−5 dB, −10 dB, −30 dB, or gap) than the baseline, at 30, 40, and 50 ms before the test stimulus, were used as prepulses. Magnetic responses to the ten types of stimuli (test stimulus alone, control, four types of tests with prepulses, and four types of prepulses alone) were recorded in 10 healthy subjects. The change-related N1m component, peaking at approximately 130 ms, and its PPI were investigated. The degree of PPI caused by the −5 dB prepulse was significantly weaker than that caused by other prepulses. The degree of PPI caused by further decreases in prepulse magnitude showed a plateau level between the −10 dB and gap prepulses. The results suggest that there is a physiologically significant range of sensory changes for PPI, which plays a role in the change detection for survival.

## 1. Introduction

For survival, one needs to focus on the most important event within a great deal of incoming sensory information; however, the mechanisms of this neural process remain unclear. One possible mechanism is a filter mechanism that protects sensory signals from the interference of subsequent sensory information for a certain period. Reflexes are involuntarily caused by sensory stimulus. The gating system has been investigated using the prepulse inhibition (PPI) of acoustic startle reflexes (ASRs), in which a weak preceding sound attenuates the following startle response (eye blink) to a louder sound [1]. The PPI of ASRs has been used in the clinical research of psychiatric disorders, particularly schizophrenia [2,3,4]. The reduction of PPI of ASR in schizophrenia is considered to reflect deficits of sensory processing relating to a flood of sensory information. The PPI of ASR is common across mammals; therefore, it has been used in genetic mouse models of schizophrenia as a translational tool [5,6].

An abrupt change in a sound feature [7], including a gap [8,9] in a continuous sound, elicits cerebral responses that can be recorded by electroencephalography (EEG) and magnetoencephalography (MEG) with high temporal resolution. The cerebral response is based on a comparison between the preceding and novel sounds with sensory memory [7,10,11,12], and it depends on the magnitude of change in the sound feature [7,13,14,15,16]. Based on these findings, the evoked response is called the change-related response. Similar to the PPI of ASR, neural inhibitory systems have been investigated by using the PPI of the change-related cerebral response. The PPI of ASR and the PPI of the change-related responses seem to indicate “sensory motor gating” and “sensory gating”, respectively. The measurement of the PPI of change-related responses has the advantage of being able to directly observe the neural inhibitory process. However, the mechanisms of the PPI of change-related responses remain unclear.

With a prepulse of greater magnitude, stronger inhibition is induced in the PPI of change-related responses [17,18], as well as that of ASRs [2]. Sound pressure decreases as well as a sound pressure increases can serve as prepulses for the PPI of change-related responses [19,20]. We recently reported that a −10 dB prepulse induced a greater PPI of change-related responses than did a −5 dB prepulse [19]. The aim of this study was to use a large range of decrease in prepulse magnitude (from−5 dB to gap) in order to investigate whether the degree of PPI is ever-increasing or reaches a plateau at a certain prepulse magnitude as the prepulse magnitude decreases from the baseline.

## 2. Materials and Methods

### 2.1. Subjects

Ten healthy male volunteers with normal hearing (28.4 ± 5.8 years) participated in this study. All subjects recruited for this study were right-handed, according to the Edinburgh Handedness Inventory [21]. The study was approved by the Clinical Research Ethics Review Committee of Mie University Hospital, Tsu, Japan and the Ethics Committee of the National Institute for Physiological Sciences, Okazaki, Japan. Written consent was obtained from all subjects after an explanation of the study.

### 2.2. Stimuli

**Figure 1:**
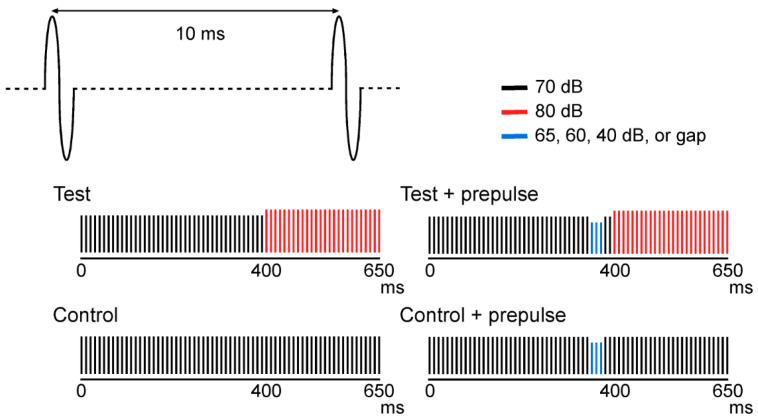
Sound stimuli used in this study. Four types of stimuli consisting of a repeated 1-ms click are illustrated.

Figure 1 shows the sound stimuli used in this study. The control stimulus was a train of 1 ms clicks at 100 Hz, 650 ms of duration, and 70 dB of sound pressure. The test stimulus was an abrupt 10 dB increase in sound pressure at 400 ms after the onset of sound. Three consecutive clicks 30, 40, and 50 ms before the abrupt increase in sound pressure were removed (gap) or made weaker than the background by 5, 10, or 30 dB. Ten stimuli (test alone, control, four types of test with prepulse, and four types of prepulse) were randomly presented through insert earphones (E-A-Rtone 3A, Aero, Indianapolis, IN, USA). The trial-to-trial interval was 900 ms.

### 2.3. MEG Recordings

Magnetic responses were recorded using a helmet-shaped 306-channel MEG system (Vector-view; ELEKTA Neuromag, Helsinki, Finland) in a silent, magnetically shielded room. During MEG recording, subjects were instructed to ignore the presented sound stimuli and watch a silent movie in front of them. MEG signals obtained from 204 planar-type gradiometers were used in this study. The bandpass filter was 0.1–330 Hz, and the sampling rate was 2000 Hz. Trials with noise larger than 3000 fT/cm were excluded from the averaging of at least 120 trials.

### 2.4. Analysis

**Figure 2:**
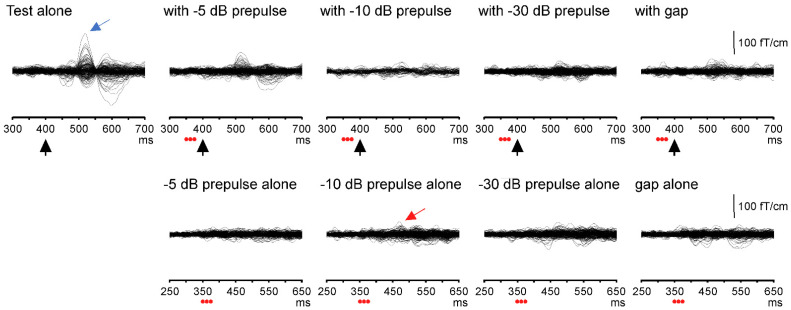
Superimposed MEG waveforms obtained from 204 gradiometers in a representative subject. Tests with/without a prepulse response are represented by the upper trace, and prepulse alone responses are represented by the lower trace. Black arrows: change onset; red circles: three click sounds as a prepulse. Blue and red arrows indicate Change-N1m evoked by the test and prepulse stimuli, respectively.

Using averaged MEG epochs for each stimulus for each subject, difference waveforms for the test alone and prepulse alone responses were obtained by subtracting waveforms for the control stimulus from those for the test alone stimulus and the four prepulse alone stimuli (−5 dB, −10 dB, −30 dB, and gap), respectively. Similarly, difference waveforms for the test with prepulse responses were obtained by subtracting waveforms for the prepulse alone waveform from those for the test with prepulse under each prepulse condition (Figure 2).

By using the brain electric source analysis (BESA) software package (GmbH, Gräfelfing, Germany), dipole analyses of change-related responses were performed using a bandpass filter of 1–35 Hz. The 100 ms period before the change onset of the test stimulus was used as a baseline. Using the difference waveform, a dipole in each hemisphere was estimated for the 20 ms time window around the Change-N1m, peaking around 130 ms after the change onset obtained from the test response for each subject. The estimated dipole model was applied to the remaining difference waveforms for each subject. To avoid an undesirable baseline shift, the Change-N1m peak amplitudes and latencies were measured using source strength waveforms. The amplitude of Change-N1m was a peak-to-peak amplitude between the Change-N1m peak and a polarity-reversed earlier peak.

Using five averaged waveforms (one test alone response and four tests with a prepulse response), as shown in Figure 2 (upper), the degree of PPI (i.e., %PPI) was calculated for each prepulse condition in each subject. The %PPI of Change-N1m was defined as (test alone response–test with a prepulse response)/test alone response × 100. The amplitude and %PPI of Change-N1m were statistically analyzed by a two-way repeated analysis of variance (ANOVA) with Hemisphere and Stimulus/Prepulse conditions as independent factors. If the sphericity condition was violated (*p* value < 0.05), Greenhouse–Geisser corrections were appropriately used. The Bonferroni–Dunn test was used for post-hoc comparison. Significance was determined by *p* values of <0.05. The data are expressed as the mean ± standard deviation, determined using the SPSS for Windows version 25 software (IBM, New York, NY, USA).

## 3. Results

**Figure 3:**
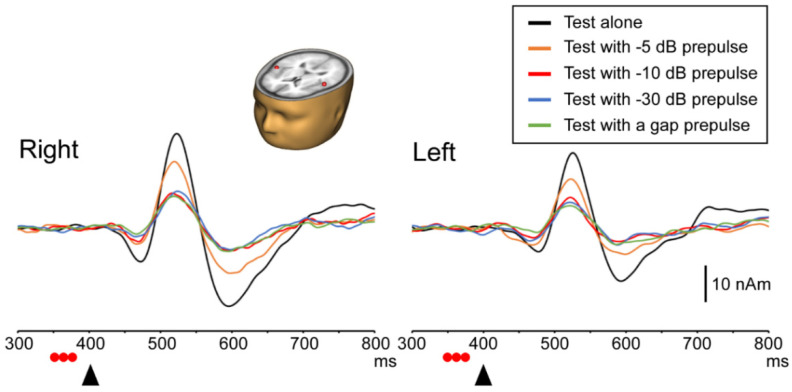
Grand-averaged source strength waveforms. Note that the amplitude of Change-N1m decreases and reaches a plateau with a decrease in prepulse sound intensity level. The mean locations of estimated current dipoles (ECDs) are overlaid on a standard MR image. The x-axis was fixed with the pre-auricular points, the positive direction being to the **right**. The positive y-axis passed through the nasion, and z-axis therefore pointed upward. The mean locations of the ECDs were 54. 0 ± 3.5, 14.6 ± 7.2, and 58.0 ± 6.9 mm for the **right** and −54.5 ± 10.2, 7.8 ± 10.0, and 63.0 ± 5.8 mm for the **left**. Black arrows: change onset; red circles: three click sounds as a prepulse.

For instructive purposes, Figure 3 shows the grand-averaged source strength waveforms and the generator of Change-N1m, which is located at the auditory cortex on both hemispheres. Table 1 summarizes the latency of Change-N1m for the test and prepulse alone responses.

### 3.1. Test Response with and without a Prepulse

**Figure 4:**
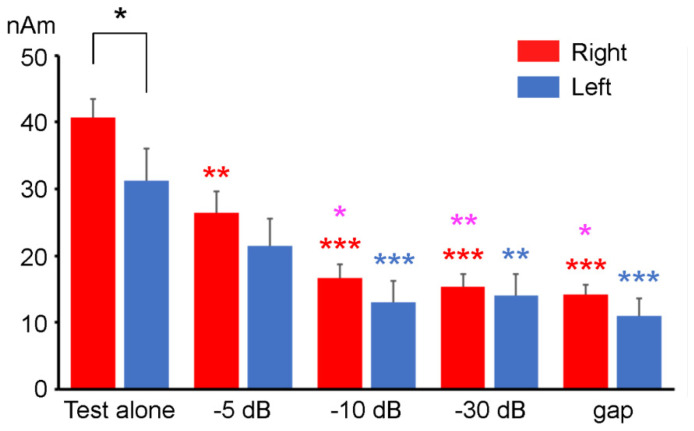
The mean amplitude of Change-N1m elicited by the test stimulus (10 dB increase from the baseline SPL) with and without a prepulse. Error bars indicate the standard errors. Asterisks indicate significant differences, as calculated with Bonferroni’s post hoc test. Asterisks colored by red, pink, and blue indicate significant differences compared to the right test alone, the right test with the −5 dB prepulse, and the left test alone, respectively. *: *p* < 0.05; **: *p* < 0.01; ***: *p* < 0.001.

The ANOVA results showed that the amplitude of Change-N1m elicited by the test stimulus significantly differed among the five stimuli (F (4, 36) = 51.85, *p* < 0.001) but not between hemispheres (F (1, 9) = 2.87, *p* = 0.12). There was a significant Stimulus × Hemisphere interaction (F (2.2, 19.85) = 3.59, *p* < 0.05). As shown in Figure 4, in the right hemisphere, the Change-N1m amplitude for the test alone response was significantly greater than those for the test with the −5 dB, −10 dB, −30 dB, and gap prepulse responses. The Change-N1m amplitude for the test with the −5 dB prepulse response was significantly higher than those for the test with the −10 dB, −30 dB, and gap prepulse responses. There were no significant differences among the −10 dB, −30 dB, and gap prepulse responses. In the left hemisphere, the Change-N1m amplitude did not significantly differ between the test alone response and the test with the −5 dB prepulse response (*p* = 0.088). The Change-N1m amplitude for the test alone response was significantly greater than other three tests with prepulse responses. There were no significant differences among the four tests with prepulse responses. Regarding laterality, the amplitude of Change-N1m in the right hemisphere was higher than that in the left only in the test alone response. Regarding the peak latency of Change-N1m, the ANOVA results showed no significant difference among the stimuli (F (4, 36) = 1.50, *p* = 0.22) and between hemispheres (F (1, 9) = 1.30, *p* = 0.28). There was also no significant interaction (F (1.84, 16.59) = 0.56, *p* = 0.57).

### 3.2. PPI

**Figure 5:**
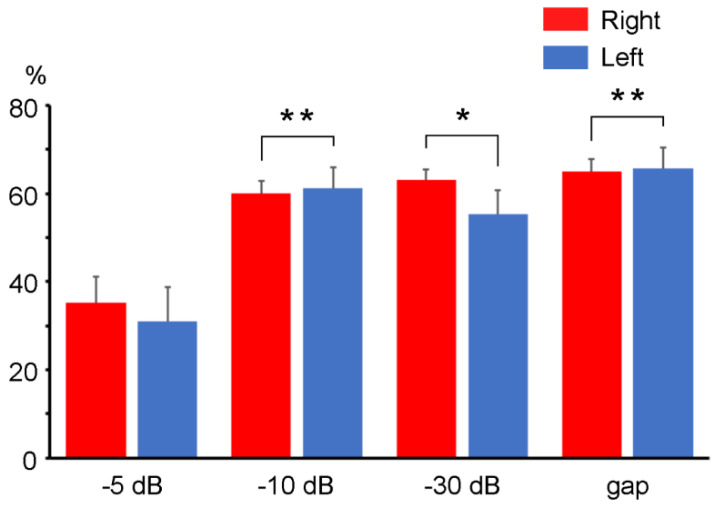
The mean suppression rate of Change-N1m. Error bars: standard mean errors. Asterisks indicate significant differences compared to the −5 dB prepulse condition, as calculated with Bonferroni’s post hoc test. *: *p* < 0.05; **: *p* < 0.01.

The degree of inhibition for the Change-N1m amplitude significantly differed among the four prepulse conditions (F (3, 27) = 19.05, *p* < 0.001) but not between hemispheres (F (1, 9) = 0.40, *p* = 0.54). There was no significant interaction (F (3, 27) = 1.03, *p* = 0.39). As shown in Figure 5, the %PPI value for the −5 dB prepulse condition was significantly smaller than those for the −10 dB, −30 dB, and gap prepulse conditions. There were no significant differences among the −10 dB, −30 dB, and gap prepulses.

### 3.3. Responses to Prepulse Alone Stimuli

**Figure 6:**
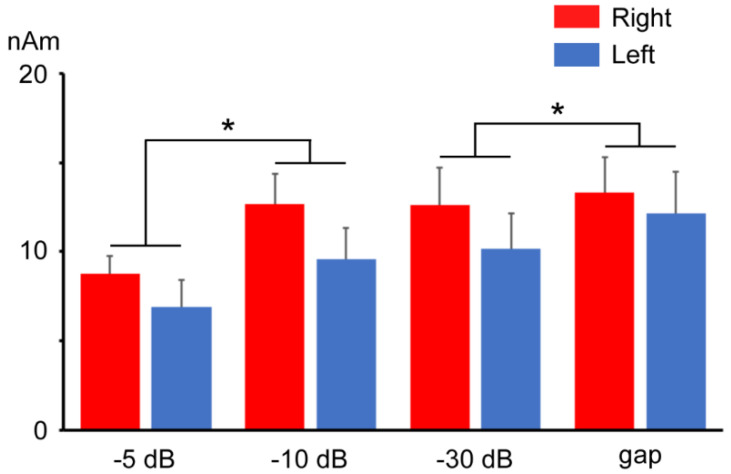
The mean amplitude of Change-N1m elicited by each prepulse. Error bars indicate the standard errors. Asterisks indicate significant differences, as calculated with Bonferroni’s post hoc test. *: *p* < 0.05.

The ANOVA results showed that the Change-N1m amplitude significantly differed among the prepulses (F (1.44, 12.95) = 6.22, *p* < 0.05) but not between hemispheres (F (1, 9) = 3.89, *p* = 0.08). There was no significant interaction (F (3, 27) = 1.03, *p* = 0.40). As shown in Figure 6, the Change-N1m amplitude for the −5 dB prepulse alone response was significantly lower than that for the −10 dB prepulse alone response and tended to be lower than that for the gap prepulse alone response (*p* = 0.081). The Change-N1m amplitude for the −30 dB prepulse response was also significantly lower than that for the gap prepulse response. Regarding the peak latency of Change-N1m in the prepulse alone response, the ANOVA results showed no significant effect of prepulses (F (1.50, 13.47) = 1.84, *p* = 0.20) and between hemispheres (F (1, 9) = 0.51, *p* = 0.50). There was also no significant interaction (F (1.38, 12.4) = 0.09, *p* = 0.84).

## 4. Discussion

As in previous studies [17,18,19,22,23,24], even when the sound feature changes of the prepulse (sound pressure decrease) differed from the following test stimulus (sound pressure increase) in our study, the change-related response to the following test stimulus was attenuated by all prepulse conditions. The inhibition by the −5 dB prepulse was weaker than that by the −10 dB prepulse, which was congruent with our previous EEG study using a similar paradigm [19]. Our main findings are as follows. As the prepulse magnitudes decreased, the degree of PPI of the change-related response showed a plateau level between the −10 dB and gap prepulse conditions. In contrast, the amplitude of Change-N1m elicited by a prepulse alone stimulus increased with the decrease of the prepulse magnitude.

Greater decreases (i.e., greater changes) of the prepulse magnitude caused increases of the change-related response itself but a plateau level of the inhibition of the subsequent change-related response. A weak change of the prepulse, which could not elicit a change-related response, also activates the inhibitory neural process [17]. Combined with the present results that showed the prompt reaching of a plateau level, we consider that the PPI of change-related responses reflects an inhibitory circuit that operates within a relatively narrow range, leading to an efficient process for the change detection in daily life. Incongruent with our results, previous studies using a subtle sound increase as a prepulse showed that the inhibition rate depended on the degree of the change of the prepulse magnitude [17,18]. This discrepancy may have been caused by the chosen types of prepulse (increase or decrease in sound pressure). The role of neural inhibition caused by the prepulse is to protect against interference with subsequent processing. Therefore, an inhibitory circuit caused by the prepulse with a sound pressure increase could operate in a wider range than that caused by the prepulse with a sound pressure decrease. This might mean that the sensory processing of increases in sound pressure is biologically more important than that of decreases in sound pressure.

In this study, the amplitude of Change-N1m for the prepulse alone stimulus was congruent with previous findings that the Change-N1m amplitude depends on the degree of change in sound pressure [7,13,14,16]. Considering the relationship with prepulse magnitude, the behavior of the amplitude of Change-N1m elicited by a prepulse and the PPI of Change-N1m elicited by a following test stimulus were clearly different. A similar discrepancy has previously been reported [25,26]. This means that the change-related response and the inhibition evoked by the prepulse reflect distinct neural processes.

The right auditory cortex is considered to play a primary role in the automatic change detection. In line with our previous MEG [17] and EEG [20] studies, the present study revealed the right-hemisphere predominance of the Change-N1m amplitude for the test alone response but not for the prepulse alone response. This might have been due to the small number (three) of clicks used as a prepulse stimulus, because the Change-N1m amplitude depends on the number of clicks with an abrupt sound pressure change in a click train sound [25]. On the other hand, the lack of hemispheric difference in the inhibition rate might indicate that the action of cortical inhibition, locally activated by GABAergic interneurons [27], is common between hemispheres.

The present study had several limitations. First, a short prepulse–test interval was used. A prepulse with a long interval (600 ms) has also been shown to attenuate the Change-N1 response [25,28]. Considering our results in combination with those a pharmacological study [27], we suggest the existence of several distinct inhibitory mechanisms. Further research is needed to confirm whether a prepulse with a long prepulse–test interval would yield similar results to those in the present study. Second, only male subjects were recruited in this study. Several studies have shown the existence of a gender difference in the PPI of ASRs. Furthermore, a previous study reported that the PPI of ASRs is affected by the menstrual cycle [29]. Thus, the gender differences need to be clarified in a further study.

A high test–retest reliability has been shown for the PPI of the change-related response [30], as well as the change-related response itself [31,32,33]. The PPI of the change-related response paradigm is useful to simultaneously evaluate sensitivity in the change detection and neural inhibitory function for clinical research. As PPI deficits of ASRs in schizophrenia have been reported [2,4], the PPI deficit of change-related responses is also expected. Recently, the PPI of ASRs in athletes was found to be greater than that in healthy controls and to correlate to scores of physical conditioning parameters [34]. A great deal of interest has been focused on neurophysiology in sports science. Investigations of the PPI of change-related responses might also play a role in this research field. The magnitude of prepulse intensity is important, so a prepulse with a ceiling effect might play a role in comparisons between groups.

## 5. Conclusions

Using a prepulse with a decrease in sound pressure from the baseline with a large range of change (from −5 dB to gap), we investigated the relationship between the magnitude of the change of the prepulse and the inhibition rate of the following change-related response in detail. The degree of PPI by the −10 dB prepulse was greater than that by the −5 dB prepulse; however, the degree of PPI showed a plateau level between the −10 dB and a gap prepulse conditions. Our results suggest a physiologically significant range for PPI, which plays a significant role in the neural circuits involved in the change detection.

## Figures and Tables

**Table 1:** Peak latency of Change-N1m.

	Test Alone	Test with a Prepulse			
		−5 dB	−10 dB	−30 dB	gap
Test response					
Right	116.2 ± 27.5	110.0 ± 25.8	116.7 ± 30.9	118.7 ± 29.1	123.4 ± 13.9
Left	126.4 ± 8.5	122.0 ± 8.4	121.4 ± 10.1	126.8 ± 16.4	125.2 ± 15.1
		**Prepulse Alone**			
		**−5 dB**	**−10 dB**	**−30 dB**	**gap**
Prepulse response					
Right		145.4 ± 25.5	127.8 ± 25.1	134.7 ± 20.0	138.2 ± 23.5
Left		151.5 ± 33.1	133.2 ± 22.1	134.5 ± 18.1	141.5 ± 23.2

Data are presented as mean ± standard deviation.

## Data Availability

The data presented in this study are available from the corresponding author on reasonable request.
